# Integrable nonlinear evolution equations in three spatial dimensions

**DOI:** 10.1098/rspa.2022.0074

**Published:** 2022-07

**Authors:** A. S. Fokas

**Affiliations:** ^1^ Department of Applied Mathematics and Theoretical Physics, University of Cambridge, Cambridge, UK; ^2^ Viterbi School of Engineering, University of Southern California,Los Angeles, CA, USA; ^3^ Mathematics Research Center, Academy of Athens, Athens, Greece

**Keywords:** integrable, nonlinear, evolution, multi-dimensional, *d*-bar

## Abstract

There are integrable nonlinear evolution equations in two spatial variables. The solution of the initial value problem of these equations necessitated the introduction of novel mathematical formalisms. Indeed, the classical Riemann–Hilbert problem used for the solution of integrable equations in one spatial variable was replaced by a non-local Riemann–Hilbert problem or, more importantly, by the so-called d-bar formalism. The construction of integrable nonlinear evolution equations in three spatial dimensions has remained the key open problem in the area of integrability. For example, the two versions of the Kadomtsev–Petviashvili (KP) equation constitute two-dimensional generalizations of the celebrated Korteweg–de Vries equation. Are there three-dimensional generalizations of the KP equations? Here, we present such equations. Furthermore, we introduce a novel non-local d-bar formalism for solving the associated initial value problem.

## 1. Introduction

The celebrated Korteweg–de Vries (KdV) and nonlinear Schrödinger (NLS) equations are prototypical integrable nonlinear evolution equations in one spatial variable. Every integrable nonlinear evolution equation in one spatial dimension has several integrable versions in two spatial dimensions. Two such physically significant generalizations of the KdV are the Kadomtsev-Petviashvili I (KPI) and II (KPII) equations. Similarly, two analogous generalizations of the NLS are the Davey–Stewartson I (DSI) and II (DSII) equations [1,2]. It is important to emphasize that integrable nonlinear evolution equations arise in a variety of physical applications. For example, in the context of fluid mechanics, the KP equations arise in the weakly nonlinear, weakly dispersive, weakly two-dimensional limit of inviscid, irrotational water waves, and in the case of KPI when the surface tension is dominant [3].

The modern history of integrable equations begins with the famous works of Martin Kruskal and his colleagues on the Cauchy problem of the KdV, using what was later called the *inverse scattering transform method* [4,5]. The next important step was taken by Peter Lax, who established that the crucial property of an integrable equation is its formulation as the *compatibility of two linear eigenvalue equations*, which were later called a Lax pair [6]. The existence of a Lax pair continues to provide the defining property of integrability [7].

The inverse scattering transform method for the solution of integrable evolution equations in 1 + 1, i.e. in one spatial and one temporal dimensions, can be considered as a *nonlinear* Fourier transform method [8]. As shown in [9], the classical Fourier transform pair and the nonlinear generalization of this pair needed for the implementation of the inverse scattering transform method can be constructed by performing the so-called *spectral analysis* of the time-independent part of the Lax pair via the formulation of a *Riemann–Hilbert problem*. This is a specific mathematical problem in the theory of *sectionally analytic functions*, namely of functions that are analytic in different sectors covering the complex plane and have given *jumps* across the curves defining these sectors [10]. The main difference between the linear and nonlinear Fourier transform pairs is the following: the construction of the classical Fourier transform pair is based on a *scalar* Riemann–Hilbert problem, which can be solved in closed form, whereas in the nonlinear case, one formulates a *matrix* Riemann–Hilbert problem, which is equivalent to a linear integral equation of the Fredholm type. Incidentally, in certain simple cases, this integral equation can be transformed to the so-called Gelfand–Levitan–Marchenko integral equation, which was used in the original integration of the KdV equation [5].

A formal method for the solution of the Cauchy problem of equations in 2 + 1, i.e. in two spatial and one temporal dimensions, was developed by Mark Ablowitz, the author of the present paper, and their collaborators. This approach was later made rigorous in several publications; see, for example, [11–17] as well as [18,19]. The extension from 1 + 1 to 2 + 1 involved the following unexpected generalization: for some equations in 2 + 1, like KPI and DSI, instead of formulating a classical Riemann–Hilbert problem, it is now required to formulate a *non-local* Riemann–Hilbert problem; for KPI and DSI this was achieved in [20] and [21], respectively. However, in general, it is necessary to go beyond the realm of analytic functions and to consider functions that are nowhere analytic in the complex plane. This leads to the formulation of the so-called d-*bar* problem [10]. Incidentally, this problem was first introduced in the field of integrability in the elegant analysis of Beals and Coifman of certain problems in 1 + 1 [22,23], although for such problems a Riemann–Hilbert formalism is not only still adequate but actually preferable to the d-bar formalism. The implementation of the d-bar method to equations in 2 + 1, where its use is indispensable, was presented in [24] for KPII and in [25] for DSII (see also [26]).

A fundamental open question in the field of integrability has been the existence of integrable evolution equations in more than two spatial dimensions. Substantial progress in this question was presented in [27], where 4 + 2 generalizations of the KP and the DS equations were presented, namely equations in *four* spatial and *two* temporal dimensions. The solution of the Cauchy problem of the 4 + 2 generalization of the KP was presented in [28]. The solution of the Cauchy problem of the 4 + 2 generalization of the DS equation was sketched in [27] and presented in detail in [29]. The solution of the Cauchy problem of the 4 + 2 generalization of the three-wave interaction equations is presented in [30]. These problems were solved using a non-local d-bar formalism, where the non-locality takes the form of a two-dimensional integral: the d-bar problem involves a two-dimensional integral over $l_{R}$ and $l_{I}$, where l is a complex variable. Hence, the nonlinear Fourier transform (spectral function) depends on four real parameters, $k_{R},k_{I},l_{R},l_{I}$, where $k_{R}$ and $k_{I}$ are the real and imaginary parts, respectively, of the spectral parameter k. This is consistent with the fact that q depends on four spatial variables.

Incidentally, another approach towards integrability in multi-dimensions was presented in [31], where it was shown that integrable nonlinear evolution equations exist in any number of dimensions; however, these equations have the major disadvantage that they involve a non-local commutator.

The questions of reducing integrable equations from 4 + 2 to equations in three dimensions and of establishing that the initial value problems of the resulting 3 + 1 equations are well-posed, and although discussed in several papers, including [32] and [33], they remain open. Here, we finally solve this problem. In particular, we solve the initial value problem of the following equation for the complex-valued function q(x,y,z,t), where x,y,z,t, are real independent variables:

1.1 $$q_{t}+\alpha q_{xxx}+\gamma \partial_{x}^{-1}(q_{yy}-q_{zz}+2iq_{yz})+\beta qq_{x}=0,\;q\in C,\;x,y,z,t\in R,$$

where α, β, γ are arbitrary real or complex constants. In the particular case when $\partial_{z}=0$ and α, β, γ are real, equation (1.1) reduces to the KP equation

1.2 $$q_{t}+\alpha q_{xxx}+\gamma \partial_{x}^{-1}q_{yy}+\beta qq_{x}=0,\;q\in R,\;x,y,t\in R.$$

Similarly, the following system of the complex-valued functions $Q_{j}(x,y,z,t)$, j=1,2, where x, y, z, t are real independent variables, provides a three-spatial-dimensions integrable generalization of the general DS-type system

1.3 $$\begin{array}{rl} & \;{\left(-1\right)}^{j-1}i\partial_{\bar{t}}Q_{j}+(\alpha \partial_{x}^{2}+\beta \partial_{\bar{\eta }}^{2})Q_{j}\\ & \;\;+\gamma Q_{j}(\delta \partial_{x}^{-1}\partial_{\bar{\eta }}+\delta^{-1}\partial_{\bar{\eta }}^{-1}\partial_{x})Q_{1}Q_{2}=0,\;j=1,2,\\ & Q_{1},Q_{2}\in C,\;\eta =y+iz,\;x,y,z,t\in R,\;\partial_{\bar{\eta }}=\frac{1}{2}(\partial_{y}+i\partial_{z})\\ \mathrm{and}\; & \;(\partial_{\bar{\eta }}^{-1}f)(y,z)=\frac{1}{\pi }\iint_{R^{2}}\frac{f(y^{\prime },z^{\prime })}{\eta -\eta^{\prime }}\;\text{d}y^{\prime }\;\text{d}z^{\prime }, \end{array}\}$$

where α β, γ, δ are arbitrary real or complex constants. In the particular case when $\partial_{z}=0$ and α, β, γ, δ are real, equations (1.3) reduce to the DS-type system

1.4 $$\begin{array}{rl} & {\left(-1\right)}^{j-1}i\partial_{t}Q_{j}+(\alpha \partial_{x}^{2}+\beta \partial_{y}^{2})Q_{j}\\ & \;+\gamma Q_{j}(\delta \partial_{x}^{-1}\partial_{y}+\delta^{-1}\partial_{y}^{-1}\partial_{x})Q_{1}Q_{2}=0,\;j=1,2\\ \mathrm{and}\; & Q_{1},Q_{2}\in C,\;x,y,t\in R, \end{array}\}$$

where we have renamed β/4 as β and δ/2 as δ. In the particular case of $Q_{1}=q$, $Q_{2}=\sigma \bar{q}$, $\sigma^{2}=1$, equations (1.4) reduce to the DS equation

1.5 $$iq_{t}+(\alpha \partial_{x}^{2}+\beta \partial_{y}^{2})q+\sigma \gamma q(\delta \partial_{x}^{-1}\partial_{y}+\delta^{-1}\partial_{y}^{-1}\partial_{x})|q{|}^{2}=0,\;q\in C,\;x,y,t\in R.$$

It is noted that equation (1.1) has the disadvantage that there does not appear to be a real reduction of it in 3 + 1 involving a *single* equation, i.e. it seems necessary to consider the system of two equations for the real and imaginary parts of q. Similarly, whereas equations (1.4) admit a reduction to the *single* DS equation (1.5), there does not appear to be a reduction to a single equation of the system in equations (1.3).

A 3 + 2 generalization of the KP equation is given by

1.6 $$q_{t}+iq_{\rho }+\alpha q_{xxx}+\gamma \partial_{x}^{-1}(q_{yy}-q_{zz}+2iq_{yz})+\beta qq_{x}=0,$$

where α, β, γ are arbitrary real or complex constants. This equation involves a second time-like variable, ρ. This variable could be interpreted, for example, as a specific quantity measuring the dependence of the underlying process on a particular property of the system.

What is the motivation for introducing a second time-like variable? It is shown in proposition 3.2 that the time dependence of the nonlinear Fourier transform (spectral function) associated with equation (1.6) involves the exponential exp⁡(iTt+iRρ), where T and R are specific real functions of the spectral variables $k_{R},k_{I},\xi$. In the linear limit, the spectral function becomes the Fourier transform of the solution q, and $k_{R},k_{I},\xi$ are the three variables in the Fourier space corresponding to the three variables, x,y,z in the physical space. The above time dependence shows that equation (1.6) is a dispersive PDE and hence its Cauchy problem is well-posed. On the other hand, the time dependence of the spectral function associated with equation (1.1) involves the exponential exp⁡(iTt−Rt), which immediately raises the question of whether this equation is well-posed. The main achievement of this work is to show that this is indeed the case: it is shown in proposition 3.5 that there is a novel d-bar formalism that ensures R is always non-negative. Hence, the underling initial value problem is well-posed. The well-posedness of equation (1.1) is consistent with the results presented in the companion paper [34], where a multitude of explicit non-singular solutions are presented, including multi-solitons, multi-breathers, and multi-rational solutions.

The implementation of the d-bar method to the system in equations (1.3), as well as to the reduced version (to three spatial dimensions) of the 4 + 2 generalization of the three-wave interaction equations, is work in progress.

## 2. Three-dimensional extensions of the Kadomtsev–Petviashvili and the Davey–Stewartson-type systems

It was shown in [27] that the complexification of the independent variables x, y, t of the KP equation yield the following 4 + 2 integrable extension of the KP equation:

2.1 $$q_{\bar{t}}=\frac{1}{4}q_{\bar{x}\bar{x}\bar{x}}-\frac{3}{2}qq_{\bar{x}}+\frac{3}{4}\partial_{\bar{x}}^{-1}q_{\bar{y}\bar{y}},$$

where

2.2 $$t=t_{1}+it_{2},\;x=x_{1}+ix_{2},\;y=y_{1}+iy_{2},\;t_{1},t_{2},x_{1},x_{2},y_{1},y_{2}\in R.$$

Equations (2.2) imply

2.3 $$\begin{array}{rl} & \partial_{\bar{t}}=\frac{1}{2}(\partial_{t_{1}}+i\partial_{t_{2}}),\;\partial_{\bar{x}}=\frac{1}{2}(\partial_{x_{1}}+i\partial_{x_{2}}),\;\partial_{\bar{y}}=\frac{1}{2}(\partial_{y_{1}}+i\partial_{y_{2}})\\ \mathrm{and}\; & (\partial_{\bar{x}}^{-1}f)(x_{1},x_{2})=\frac{1}{\pi }\iint_{R^{2}}\frac{f(x_{1}^{\prime },x_{2}^{\prime })}{x-x^{\prime }}\;\text{d}x_{1}^{\prime }\;\text{d}x_{2}^{\prime }. \end{array}\}$$

It is important to emphasize that equation (2.1) is a genuine equation in 4 + 2, namely the dependent variable $q(x_{1},x_{2},y_{1},y_{2},t_{1},t_{2})$ depends on the four spatial variables $x_{1}$, $x_{2}$, $y_{1}$, $y_{2}$ and on the two temporal variables $t_{1}$, $t_{2}$.

Letting

2.4 $$\xi =ax_{1}+bx_{2},\;\tau =\tilde{a}t_{1}+\tilde{b}t_{2},\;a,b,\tilde{a},\tilde{b}\;\text{are real constants},$$

we find

2.5 $$\partial_{\bar{x}}=A\frac{\partial }{\partial_{\xi }}\;\mathrm{and}\;\partial_{\bar{t}}=\tilde{A}\frac{\partial }{\partial_{\tau }},\;A=\frac{a+ib}{2},\;\tilde{A}=\frac{\tilde{a}+i\tilde{b}}{2}.$$

Hence, equation (2.1) becomes

$$\tilde{A}q_{\tau }-\frac{A^{3}}{4}q_{\xi \xi \xi }+\frac{3}{2}Aqq_{\xi }-\frac{3}{4}\frac{1}{A}\partial_{\xi }^{-1}q_{\bar{y}\bar{y}}=0.$$

Replacing q by Qq, y by $y/\bar{Y}$, where Q and Y are complex constants, we find

2.6 $$q_{\bar{\tau }}+\alpha q_{\xi \xi \xi }+\beta qq_{\xi }+\gamma \partial_{\xi }^{-1}q_{\bar{y}\bar{y}}=0,\;\alpha ,\beta ,\gamma \in C,$$

where

2.7a $$\alpha =-\frac{1}{4}\frac{A^{3}}{\tilde{A}},\;\beta =\frac{3}{2}\frac{AQ}{\tilde{A}}\;\mathrm{and}\;\gamma =-\frac{3}{4}\frac{Y^{2}}{A\tilde{A}}.$$

Equation (2.6) with $\tilde{a}t_{1}=2t,\;t_{2}=0$ is equation (1.1), whereas equation (2.6) with $\tilde{a}t_{1}=2t,\;\tilde{b}t_{1}=2\rho$ is equation (1.6).

Equations (2.7*a*) imply

2.7b $$\tilde{A}=-\frac{A^{3}}{4\alpha },\;Q=-\frac{A^{2}\beta }{6\alpha }\;\mathrm{and}\;Y^{2}=\frac{A^{4}\gamma }{3\alpha }.$$

The t-independent part of the Lax pair of equation (2.1), after the change of variables in equations (2.4), becomes

$$[\partial_{\bar{y}}+k^{2}-{\left(A\partial_{\xi }+k\right)}^{2}+q]\mu =0.$$

Thus, the t-independent part of the Lax pair of equation (2.6) is

$$\left[Y\partial_{\bar{y}}+k^{2}-A^{2}{\left(\partial_{\xi }+\frac{k}{A}\right)}^{2}+Qq\right]\mu =0.$$

Replacing k with Ak and simplifying using equations (2.7*b*), we find

2.8 $$\left[{\left(\frac{\gamma }{3\alpha }\right)}^{\left(1/2\right)}\partial_{\bar{y}}-\partial_{\xi }^{2}-2k\partial_{\xi }-\frac{\beta }{6\alpha }q\right]\mu =0.$$

Similarly, the t-part of the Lax pair of equation (2.1), after the change of variables in equations (2.4), yields

2.9 $$\left\{\partial_{\bar{\tau }}-4\alpha k^{3}+4\alpha {\left(\partial_{\xi }+k\right)}^{3}+\beta q(\partial_{\xi }+k)+\frac{\beta }{2}\left[q_{\xi }+{\left(\frac{\gamma }{3\alpha }\right)}^{\left(1/2\right)}\partial_{\xi }^{-1}q_{\bar{y}}\right]\right\}\mu =0.$$

Renaming τ, ξ, $y_{1}$, $y_{2}$ as t, x, y, z, and renaming γ/4 as γ, we find the following result: the integrable 3 + 1 equation (1.1) possesses the Lax pair

2.10 $$\left[{\left(\frac{\gamma }{3\alpha }\right)}^{\left(1/2\right)}(\partial_{y}+i\partial_{z})-\partial_{x}^{2}-2k\partial_{x}-\frac{\beta }{6\alpha }q\right]\mu =0$$

and

2.11 $$\left\{\partial_{t}+4\alpha (\partial_{x}^{3}+3k\partial_{x}^{2}+3k^{2}\partial_{x})+\beta q(\partial_{x}+k)+\frac{\beta }{2}\left[q_{x}+{\left(\frac{\gamma }{3\alpha }\right)}^{\left(1/2\right)}\partial_{x}^{-1}(q_{y}+iq_{z})\right]\right\}\mu =0.$$

Similarly, equation (1.6) possesses the Lax pair formed by equation (2.10) and by the equation formed from equation (2.11) by replacing $\partial_{t}$ with $\partial_{t}+\text{i}\partial_{\rho }$.

We next consider the 2 + 1 version of the DS type system

2.12 $$\begin{array}{rl} & {\left(-1\right)}^{j-1}\partial_{t}Q_{j}+\frac{1}{4}(\partial_{x}^{2}+\partial_{\eta }^{2})Q_{j}-\frac{1}{2}Q_{j}(\partial_{x}^{-1}\partial_{\eta }+\partial_{\eta }^{-1}\partial_{x})Q_{1}Q_{2}=0,\;j=1,2\\ \mathrm{and}\; & Q_{1},Q_{2}\in C,\;x,\eta ,t\in R. \end{array}\}$$

The complexification of the independent variables

2.13 $$t=t_{1}+it_{2},\;x=x_{1}+ix_{2}\;\mathrm{and}\;\eta =\eta_{1}+i\eta_{2},\;t_{1},t_{2},x_{1},x_{2},\eta_{1},\eta_{2}\in R$$

yields the 4 + 2 integrable system

2.14 $${\left(-1\right)}^{j-1}\partial_{\bar{t}}Q_{j}+\frac{1}{4}(\partial_{\bar{x}}^{2}+\partial_{\bar{\eta }}^{2})Q_{j}-\frac{1}{2}Q_{j}(\partial_{\bar{x}}^{-1}\partial_{\bar{\eta }}+\partial_{\bar{\eta }}^{-1}\partial_{\bar{x}})Q_{1}Q_{2}=0,\;j=1,2,$$

where

2.15 $$\partial_{\bar{\eta }}=\frac{1}{2}(\partial_{\eta_{1}}+i\partial_{\eta_{2}}),\;(\partial_{\bar{\eta }}^{-1}f)(\eta_{1},\eta_{2})=\frac{1}{\pi }\iint_{R^{2}}\frac{f(\eta_{1}^{\prime },\eta_{2}^{\prime })}{\eta -\eta^{\prime }}\;\text{d}\eta_{1}^{\prime }\;\text{d}\eta_{2}^{\prime },$$

and similarly for $\partial_{\bar{x}}$, $\partial_{\bar{x}}^{-1}$. It should be noted that there are typos for this system in [27], which are corrected in [29].

Using the change of variables in equations (2.4), proceeding as with the KP equation and finally renaming τ, ξ, $\eta_{1}$, $\eta_{2}$ as t, x, y, z, we find equations (1.3), where

$$\alpha =\frac{iA^{2}}{4\tilde{A}},\;\beta =\frac{iY^{2}}{4\tilde{A}}\;\mathrm{and}\;\delta =\frac{A^{-1}Y}{\tilde{A}},$$

and the parameter γ is generated by the scaling of $Q_{1}$ and $Q_{2}$.

## 3. The Cauchy problem for a three-dimensional extension of the Kadomtsev–Petviashvili

For simplicity of notation, we suppress the dependence on $\bar{k}$, namely instead of $F(k,\bar{k}),$ we write F(k). Thus, F(k) means that F depends on $\left(k_{R},k_{I}\right)$ instead of $k_{R}+\text{i}k_{I}$, where $k_{R}$ and $k_{I}$ denote the real and the imaginary parts, respectively, of k.

### (a) Spectral analysis of the t-independent part of the Lax pair

In what follows, instead of analysing equation (2.10), we will analyse the equation

3.1 $$[\partial_{y}+i\partial_{z}-\partial_{x}^{2}-2k\partial_{x}+q(x,y,z)]\mu (x,y,z,k)=0,\;x,y,z\in R,\;k\in C.$$

If γ/α is real, then equation (3.1) can be mapped to equation (2.10) by replacing y and z with ${\left(\gamma /3\alpha \right)}^{1/2}y$ and ${\left(\gamma /3\alpha \right)}^{1/2}z$, respectively, and by rescaling q. If γ/α is complex, then equation (3.1) can also be mapped to equation (2.10) with appropriate scaling of y and z.

We assume that q vanishes for large values of x,y,z. In what follows, we will show that a solution of equation (3.1), which tends to 1 for large values of x,y,z, satisfies the following linear integral equation:

3.2 $$\mu (x,y,z,k)=1+\int_{R^{3}}G(x-x^{\prime },y-y^{\prime },z-z^{\prime },k)\;q(x^{\prime },y^{\prime },z^{\prime })\mu (x^{\prime },y^{\prime },z^{\prime },k)\;dx^{\prime }\;dy^{\prime }\;dz^{\prime },$$

where

3.3 $$G(x,y,z,k)=-\frac{1}{{\left(2\pi \right)}^{3}}\int_{R^{3}}\frac{e^{i(\xi x+\eta y+\zeta z)}}{\xi^{2}-2ik\xi +i\eta -\zeta }\;d\xi \;d\eta \;d\zeta .$$

Furthermore, $\partial \mu /\partial \bar{k}$ satisfies the linear integral equation

3.4 $$\begin{array}{rl} \frac{\partial \mu }{\partial \bar{k}}(x,y,z,k) & =F(x,y,z,k)+\int_{R^{3}}G(x-x^{\prime },y-y^{\prime },z-z^{\prime },k)q(x^{\prime },y^{\prime },z^{\prime })\\ & \;\times \frac{\partial \mu }{\partial \bar{k}}(x^{\prime },y^{\prime },z^{\prime },k)\;dx^{\prime }\;dy^{\prime }\;dz^{\prime }, \end{array}$$

where F is defined by

3.5 $$F(x,y,z,k)=\int_{R^{3}}\frac{\partial G}{\partial \bar{k}}(x-x^{\prime },y-y^{\prime },z-z^{\prime },k)q(x^{\prime },y^{\prime },z^{\prime })\mu (x^{\prime },y^{\prime },z^{\prime },k)\;dx^{\prime }\;dy^{\prime }\;dz^{\prime }$$

and

3.6 $$\frac{\partial G}{\partial \bar{k}}(x,y,z,k)=\frac{1}{{2i\pi }^{3}}\int_{R}\xi \;e^{i[\xi x+2k_{R}\xi y+{(\xi }^{2}+2i\xi k_{I})z]\;}\;d\xi .$$

Indeed, we define the Green’s function G by

3.7 $$G_{y}+iG_{z}-G_{xx}-2kG_{x}=-\delta (x)\delta (y)\delta (z).$$

Using the identity

3.8 $$\delta (x)\delta (y)\delta (z)=\frac{1}{{\left(2\pi \right)}^{3}}\int_{R^{3}}e^{i(\xi x+\eta y+\zeta z)}\;d\xi \;d\eta \;d\zeta$$

and looking for a solution of equation (3.7) in the form

$$G=-\frac{1}{{\left(2\pi \right)}^{3}}\int_{R^{3}}g(\xi ,\eta ,\zeta ,k)\;e^{i(\xi x+\eta y+\zeta z)}\;d\xi \;d\eta \;d\zeta ,$$

we find equation (3.3).

In order to compute $\partial G/\partial \bar{k}$, we note that

$$\xi^{2}-2ik\xi +i\eta -\zeta =-2i\left\{k-\left[\frac{\eta }{2\xi }+\frac{i}{2\xi }(\zeta -\xi^{2})\right]\right\}.$$

This equation together with the identity

3.9 $$\frac{\partial }{\partial \bar{k}}\left(\frac{1}{k-\tilde{k}}\right)=\pi \delta (k_{R}-{\tilde{k}}_{R})\delta (k_{I}-{\tilde{k}}_{I}),\;k=k_{R}+{ik}_{I},\;\tilde{k}={\tilde{k}}_{R}+{i\tilde{k}}_{I},$$

imply the formula

$$\frac{\partial G}{\partial \bar{k}}=-\frac{1}{{\left(2\pi \right)}^{3}}\frac{\pi }{\left(-2i\right)}\int_{R}e^{i(\xi x+\eta y+\zeta z)}{\frac{|2\xi {|}^{2}}{\xi }|}_{\begin{array}{c} \eta =2\xi k_{R}\\ \zeta =\xi^{2}+2k_{I}\xi \end{array}}d\xi ,$$

which is equation (3.6).

Putting equation (3.6) into equation (3.5), we find

3.10 $$F(x,y,z,k)=\int_{R}\xi \hat{q}(\xi ,k)\;e^{i[\xi x+2k_{R}\xi y+(\xi^{2}+2\xi k_{I})z]}\;d\xi ,$$

where $\hat{q}(\xi ,k)$ is defined by

3.11 $$\hat{q}(\xi ,k)=\frac{1}{{16i\pi }^{2}}\int_{R^{3}}e^{-i[\xi x^{\prime }+2k_{R}\xi y^{\prime }+(\xi^{2}+2\xi k_{I})z^{\prime }]}q(x^{\prime },y^{\prime },z^{\prime })\mu (x^{\prime },y^{\prime },z^{\prime },k)\;dx^{\prime }\;dy^{\prime }\;dz^{\prime }.$$

It turns out that the functions μ and $\partial \mu /\partial \bar{k}$ satisfy the following relationship:

3.12 $$\frac{\partial \mu (x,y,z,k)}{\partial \bar{k}}=\int_{R}\xi \;e^{i[\xi x+2k_{R}\xi y+(\xi^{2}+2\xi k_{I})z]}\hat{q}(\xi ,k)\mu (x,y,z,k+i\xi )\;d\xi .$$

Indeed, the product of the exponential and the function μ(x,y,z,k+iξ) appearing in the above integral satisfy the following equation:

3.13 $$\begin{array}{rl} & e^{i[\xi x+2k_{R}\xi y+(\xi^{2}+2\xi k_{I})z]}\mu (x,y,z,k+i\xi )=e^{i[\xi x+2k_{R}\xi y+(\xi^{2}+2\xi k_{I})z]}\\ & \;+\int_{R^{3}}\hat{G}(x-x^{\prime },y-y^{\prime },z-z^{\prime },k,\xi )q(x^{\prime },y^{\prime },z^{\prime })\;e^{i[\xi x^{\prime }+2k_{R}\xi y^{\prime }+(\xi^{2}+2\xi k_{I})z^{\prime }]}\\ & \;\times \mu (x^{\prime },y^{\prime },z^{\prime },k+i\xi )\;dx^{\prime }\;dy^{\prime }\;dz^{\prime }, \end{array}$$

where

3.14 $$\hat{G}(x,y,z,k,\xi )=-\frac{1}{{\left(2\pi \right)}^{3}}\int_{R^{3}}\frac{e^{i[(\tilde{\xi }+\xi )x+(\tilde{\eta }+2k_{R}\xi )y+(\tilde{\zeta }+\xi^{2}+2\xi k_{I})z]}}{{\tilde{\xi }}^{2}-2ik\tilde{\xi }+i\tilde{\eta }-\tilde{\zeta }+2\xi \tilde{\xi }}\;d\tilde{\xi }\;d\tilde{\eta }\;d\tilde{\zeta }.$$

Letting

$$\overset{ˇ}{\xi }=\tilde{\xi }+\xi ,\;\overset{ˇ}{\eta }=\tilde{\eta }+2k_{R}\xi \;\mathrm{and}\;\overset{ˇ}{\zeta }=\tilde{\zeta }+\xi^{2}+2\xi k_{I},$$

we find

3.15 $$\hat{G}(x,y,z,k,\xi )=-\frac{1}{{\left(2\pi \right)}^{3}}\int_{R^{3}}\frac{e^{i[\overset{ˇ}{\xi }x+\overset{ˇ}{\eta }y+\overset{ˇ}{\zeta }z]}}{{\overset{ˇ}{\xi }}^{2}-2ik\overset{ˇ}{\xi }+i\overset{ˇ}{\eta }-\overset{ˇ}{\zeta }}\;d\overset{ˇ}{\xi }\;d\overset{ˇ}{\eta }\;d\overset{ˇ}{\zeta }=G(x,y,z,k).$$

In equation (3.13), replacing $\hat{G}$ with $G(x-x^{\prime },y-y^{\prime },z-z^{\prime },k)$, multiplying the resulting equation by $\xi \hat{q}(\xi ,k)$, and integrating over ξ, we find an equation identical to equation (3.5).

Equation (3.12), together with the boundary condition that μ(x,y,z,t,k) tends to 1 for large values of k, define a classical d-bar problem [7]. The solution of this problem is given by

3.16 $$\begin{array}{rl} & \mu (x,y,z,k)=1-\frac{1}{\pi }\int_{R^{3}}\frac{{\xi e}^{i[\xi x+2l_{R}\xi y+(\xi^{2}+2\xi l_{I})z]}}{l-k}\hat{q}(\xi ,l)\mu (x,y,z,l+i\xi )d\xi \;dl_{R}\;dl_{I}\\ \;\mathrm{and}\; & k\in \;C,\;l=l_{R}+il_{I}. \end{array}\}$$

This equation implies the expansion

3.17 $$\mu (x,y,z,k)=1+\frac{\mu_{1}(x,y,z)}{k}+O\left(\frac{1}{k^{2}}\right),\;k\to \infty ,$$

where $\mu_{1}(x,y,z,t)$ can be explicitly computed in terms of the numerator of the integrand of the right-hand side of equation (3.16). On the other hand, putting the expansion of equation (3.17) into equation (3.1), we find

3.18 $$q(x,y,z)=2{{(\mu }_{1}(x,y,z))}_{x}.$$

Hence,

3.19 $$q(x,y,z)=\frac{2}{\pi }\int_{R^{3}}{\xi e}^{i[\xi x+2l_{R}\xi y+(\xi^{2}+2\xi l_{I})z]}\hat{q}(\xi ,l)\mu (x,y,z,l+i\xi )\;d\xi \;dl_{R}\;dl_{I}.$$

In summary, the spectral analysis of equation (3.1) gives rise to the following nonlinear Fourier transform pair: the direct transform is given by equation (3.11), with μ defined in terms of q by equation (3.2), whereas the inverse transform is given by equation (3.19), with μ defined in terms of $\hat{q}$ by equations (3.16). In the linear limit of q, μ tends to 1 and the above transform pair becomes

3.20 $$\begin{array}{rl} & \hat{q}(\xi ,k)=\frac{1}{16{i\pi }^{2}}\int_{R^{3}}e^{-i[\xi x+2k_{R}\xi y+(\xi^{2}+2\xi k_{I})z]}q(x,y,z)\;dx\;dy\;dz\\ \mathrm{and}\; & q(x,y,z)=\frac{2}{\pi }\int_{R^{3}}{\xi e}^{i[\xi x+2k_{R}\xi y+(\xi^{2}+2\xi k_{I})z]}\hat{q}(\xi ,k)\;\;d\xi \;dk_{R}\;dk_{I}. \end{array}\}$$

The change of variables

$$\xi =k_{1},\;2k_{R}\xi =k_{2}\;\mathrm{and}\;\xi^{2}+2\xi k_{I}=k_{3}$$

maps the transform pair in equations (3.20) to the usual three-dimensional Fourier transform pair.

The above analysis provides the basis for the following result:

**Proposition 3.1.**

*Let* $q(x,y,z)\in S(R^{3})$, *where* S *denotes the space of Schwartz functions and suppose that the* $L_{1}$ *and* $L_{2}$ *norms of* q *are sufficiently small*.

*Let* $\mu (x,y,z,k),(x,y,z)\in R^{3},\;k\in C$, *be the unique solution of equation (3.2). A nonlinear Fourier transform of* q, *denoted by* $\hat{q}(\xi ,k),\;\xi \in R,\;k\in C$, *is defined by the right-hand side of equation (3.11)*.

Given $\hat{q}(\xi ,k)$, let μ(x,y,z,k) be the unique solution of equation (3.16). Let q be defined by the right-hand side of equation (3.16). Then q constitutes the inverse of the nonlinear Fourier transform $\hat{q}$.

**Proof.**

The result stated in proposition 3.1 is derived earlier. It is straightforward to make this formalism rigorous under the assumption that the $L_{1}$ and $L_{2}$ norms of q are sufficiently small. Indeed, in this case, both the linear integral equations (3.2) and (3.16) have unique solutions. The easiest way to obtain these results is to use the fact that for a ‘small q’, the above formalism reduces to the formulae for the direct and inverse Fourier transform pair, which are well-defined if the $L_{1}$ and $L_{2}$ norms of q exist.

As it is well-known, the inverse scattering transform method, as well as its d-bar generalization, are based on the spectral analysis of the t-independent part of the Lax pair. Different time evolutions impose different time dependence on the associated nonlinear Fourier transform. In our case, equations (1.1) and (1.6) impose different time dependence on $\hat{q}(\xi ,k)$. We first consider equation (1.6).

### (b) Solution of the Cauchy problem of equation (1.6)

**Proposition 3.2.**

*Given the complex-valued function* $q_{0}(x,y,z)\in S(R^{3})$, *whose* $L_{1}$ *and* $L_{2}$ *norms are sufficiently small, let* $\mu_{0}(x,y,z,k)$ *denote the solution of equation (3.2), where* q *is replaced by* $q_{0}$. *Let* ${\hat{q}}_{0}(\xi ,k)$ *be defined by the right-hand side of equation (3.11), with* q *and* μ *replaced by* $q_{0}$ *and* $\mu_{0}$, *respectively. Let* μ(x,y,z,t,ρ,k) *be the solution of equation (3.16), with* $\hat{q}(\xi ,k)$ *replaced by*

3.21 $$\begin{array}{rl} & \hat{q}(\xi ,k,t,\rho )={\hat{q}}_{0}(\xi ,k)\;e^{i(Tt+R\rho )},\;T=-\xi^{3}+3\xi (k_{R}^{2}-k_{I}^{2})-3\xi^{2}k_{I}\\ \mathrm{and}\; & R=3\xi k_{R}(\xi +2k_{I}). \end{array}\}$$

Let q(x,y,z,t,ρ) be defined by the right-hand side of equation (3.19), with $\hat{q}(\xi ,k)$ and μ(x,y,z,k) replaced by $\hat{q}(\xi ,k,t,\rho )$ and μ(x,y,z,t,ρ,k), respectively. Then q(x,y,z,t,ρ) satisfies

3.22 $$q_{t}+iq_{\rho }-\frac{1}{4}q_{xxx}+\frac{3}{4}\partial_{x}^{-1}(q_{zz}-q_{yy}-2iq_{yz})+\frac{3}{2}q_{x}q=0,$$

with

3.23 $$q(x,y,z,0,0)=q_{0}(x,y,z).$$

**Proof.**

The equation satisfied by μ(x,y,z,t,ρ,k) is equivalent to the d-bar problem

3.24a $$\frac{\partial \mu (k)}{\partial \bar{k}}=\int_{R}E(k,\xi )\mu (k+i\xi )\xi {\hat{q}}_{0}(\xi ,k)\;d\xi$$

and

3.24b $$\mu (k)=1+\frac{\mu_{1}}{k}+O\left(\frac{1}{k^{2}}\right),\;|k|\to \infty ,$$

where

3.25 $$E(k,\xi )=e^{i\xi x+i(k+\bar{k})\xi y+[i\xi^{2}+\xi (k-\bar{k})]z+iTt+iR\rho },$$

and for convenience of notation, we have suppressed the x,y,z,t,ρ dependence of μ and E.

In what follows, using the dressing method, we will show directly that if μ is defined by equations (3.24) and if q is defined in terms of μ via

3.26 $$q(x,y,z,t,\rho )=2({\mu_{1}(x,y,z,t,\rho ))}_{x},$$

or equivalently via equation (3.19), with $\hat{q}(\xi ,k)$ and μ(x,y,z,k) replaced by $\hat{q}(\xi ,k,t,\rho )$ and μ(x,y,z,t,ρ,k), respectively, then q solves equation (3.22). For this purpose, we introduce the operators

3.27 $$D_{x}=\partial_{x}+k\;\mathrm{and}\;D=\partial_{y}+i\partial_{z}+k^{2}.$$

The crucial property of these operators is that they ‘commute’ with the d-bar operator defined in equation (3.24*a*). In particular,

3.28 $$\frac{\partial }{\partial \bar{k}}{(D}_{x}\mu (k))=\int_{R}E(k,\xi )D_{x}\mu (k+i\xi )\;d\nu ,\;d\nu =\xi {\hat{q}}_{0}(\xi ,k)\;d\xi .$$

Indeed, differentiating equation (3.24*a*) with respect to x, we find

$$\frac{\partial }{\partial \bar{k}}\mu_{x}(k)=\int_{R}E(k,\xi )[\mu_{x}(k+i\xi )+i\xi \mu (k+i\xi )]d\nu .$$

Also,

$$\frac{\partial }{\partial \bar{k}}(k\mu )(k)=\int_{R}E(k,\xi )k\mu (k+i\xi )\;d\nu .$$

Adding these two equations, we find equation (3.28). Similar considerations are valid for D. Also,

$$\frac{\partial }{\partial \bar{k}}(f\mu )(k)=\int_{R}E(k,\xi )\;(q\mu )(k+i\xi )\;d\nu .$$

Hence, the function $L_{1}(k)$, defined by

3.29 $$L_{1}(k)=(D-D_{x}^{2}+q)\mu ,$$

where again we have suppressed the x,y,z,t,ρ dependence of $L_{1}(k)$, satisfies equation (3.24*a*). Hence, $L_{1}$ vanishes provided that $L_{1}(k)=O(1/k)$, |k|→∞. This is indeed the case provided that q is defined by equation (3.26).

The equation $L_{1}(k)=0$ is equation (3.1) with μ and q replaced by μ(x,y,z,t,ρ,k) and q(x,y,t,ρ), respectively. This equation provides the (t,ρ)-independent part of the Lax pair of equation (3.22). In order to define the (t,ρ)-dependent part, we introduce the operator

3.30 $$D_{\tau }=\partial_{t}+i\partial_{\rho }+k^{3}.$$

It is straightforward to verify that $D_{\tau }$ also commutes with the d-bar operator defined in equation (3.24*a*). This fact is a direct consequence of the identity

$$iT-R+k^{3}={\left(k+i\xi \right)}^{3}.$$

Since $D_{\tau }-D_{x}^{3}$ is of the order k as |k|→∞, we define $L_{2}(k)$ by

$$L_{2}(k)=(D_{\tau }-D_{x}^{3}+Q_{1}D_{x}+Q_{2})\mu ,$$

where $Q_{1}$ and $Q_{2}$ are functions of x,y,z,t,ρ, chosen by the requirement that $L_{2}(k)=O(1/k)$ as |k|→∞. Hence, the (t,ρ)-dependent part of the Lax pair is

3.31a $$\mu_{t}+i\mu_{\rho }-\mu_{xxx}-3k^{2}\mu_{x}-3k\mu_{xx}+Q_{1}(\mu_{x}+k\mu )+Q_{2}\mu =0,$$

where

3.32a $$Q_{1}=3\mu_{1x}=\frac{3}{2}q\;\mathrm{and}\;Q_{2}=3\mu_{2x}+3\mu_{1xx}-Q_{1}\mu_{1}.$$

The terms of O(1/k) of the equation $L_{1}(k)=0$ imply

3.31b $$\mu_{1y}+i\mu_{1z}-\mu_{1xx}-2\mu_{2x}+q\mu_{1}=0.$$

Thus, the second of equations (3.32*a*) yields

3.32b $$Q_{2}=\frac{3}{2}(\mu_{1xx}+\mu_{1y}+i\mu_{1z})=\frac{3}{4}(q_{x}+{\partial_{x}^{-1}q}_{y}+{i\partial_{x}^{-1}q}_{z}).$$

Either the compatibility of the Lax pair, or the terms of order 1/k of equation (3.31*a*), yield equation (3.22).

**Remark 3.3.**

A simple computation shows that, as expected, the exponential $\exp[\text{i}\xi x+2\text{i}k_{R}\xi y+\text{i}(\xi^{2}+2\xi k_{I})z+\text{i}Tt+\text{i}R\rho ]$, with T and R defined in equations (3.21), satisfies the linear version of equation (3.22) (this equation can be solved numerically by letting q=u+iv, and then solving the system of the two resulting equations).

**Remark 3.4.**

If the initial condition $q_{0}$ satisfies the relation

3.33a $$\bar{q_{0}(x,y,z)}=q_{0}(x,y,-z),$$

then, the solution of equation (3.22) satisfies the relation

3.33b $$\bar{q(x,y,z,t,\rho )}=q(x,y,-z,t,-\rho ).$$

Indeed, using the transformations ξ→−ξ, η→−η in equation (3.3), we find

$$\bar{G(x,y,z,k)}=G(x,y,-z,\bar{k}).$$

Then, equation (3.2) together with the assumption on $q_{0}$ imply

$$\bar{\mu (x,y,z,k)}=\mu (x,y,-z,\bar{k}).$$

Then, the definition of $\hat{q}(\xi ,k)$ implies

$$\bar{\hat{q}(\xi ,k)}=-\hat{q}(-\xi ,\bar{k}).$$

The definition of μ, namely equation (3.24), implies

$$\bar{\mu (x,y,z,t,\rho ,k)}=\mu (x,y,-z,t,-\rho ,\bar{k})$$

and then the definition of q implies that q satisfies equation (3.33*b*).

### (c) Solving equation (1.1)

Following an analysis similar to that presented in proposition 3.2, it follows that now, instead of equation (3.21), we have

3.34 $$\hat{q}(\xi ,k,t)={\hat{q}}_{0}(\xi ,k)\;e^{iTt-4k_{R}\xi (\xi +2k_{I})t},$$

where T is defined in equation (3.21). This means that the integral equation defining μ(x,y,z,t,k,ξ) is well-defined only if we can ensure that

3.35 $$k_{R}\xi (\xi +2k_{I})\geq 0.$$

For this purpose, since we need to control the sign of $\xi +2k_{I}$, we write the double integral over $\text{d}\xi \;\text{d}k_{I}$ in the form

$$\int_{-\infty }^{0}\;dk_{I}\int_{-\infty }^{-2k_{I}}\;d\xi +\int_{0}^{\infty }\;dk_{I}\int_{-\infty }^{-2k_{I}}\;d\xi +\int_{-\infty }^{0}\;dk_{I}\int_{-2k_{I}}^{\infty }\;d\xi +\int_{0}^{\infty }\;dk_{I}\int_{-2k_{I}}^{\infty }\;d\xi .$$

Furthermore, since we also need to control the sign of ξ, we split the first and fourth integrals as follows:

3.36 $$\begin{array}{rl} & \int_{-\infty }^{0}\;dk_{I}\int_{-\infty }^{0}\;d\xi +\int_{-\infty }^{0}\;dk_{I}\int_{0}^{-2k_{I}}\;d\xi +\int_{0}^{\infty }\;dk_{I}\int_{-\infty }^{-2k_{I}}\;d\xi \\ & \;+\int_{-\infty }^{0}\;dk_{I}\int_{-2k_{I}}^{\infty }\;d\xi +\int_{0}^{\infty }\;dk_{I}\int_{-2k_{I}}^{0}\;d\xi +\int_{0}^{\infty }\;dk_{I}\int_{0}^{\infty }\;d\xi . \end{array}$$

In the first three integrals in equation (3.36), $\xi +2k_{I}\leq 0$, thus in these integrals, we need $k_{R}\xi \leq 0$; hence, in the first integral, $k_{R}\geq 0$, in the second, $k_{R}\leq 0$, and in the third, $k_{R}\geq 0$. Similarly, in the last three integrals in equation (3.36), $\xi +2k_{I}\geq 0$, thus in these integrals, we need $k_{R}\xi \geq 0$; hence, in the fourth integral, $k_{R}\geq 0$, in the fifth, $k_{R}\leq 0$, and in the sixth, $k_{R}\geq 0$. Hence, if we define the operator $L_{k_{R},k_{I},\xi }$ by

3.37 $$\begin{array}{rl} L_{k_{R},k_{I},\xi }[\cdot ] & =\int_{0}^{\infty }\;dk_{R}\left[\int_{-\infty }^{0}\;dk_{I}\left(\int_{-\infty }^{0}\;d\xi +\int_{-2k_{I}}^{\infty }\;d\xi \right)+\int_{0}^{\infty }\;dk_{I}\left(\int_{-\infty }^{-2k_{I}}\;d\xi +\int_{0}^{\infty }\;d\xi \right)\right]\\ & \;+\int_{-\infty }^{0}\;dk_{R}\left(\int_{-\infty }^{0}\;dk_{I}\int_{0}^{-2k_{I}}\;d\xi +\int_{0}^{\infty }\;dk_{I}\int_{-2k_{I}}^{0}\;d\xi \right)[\cdot ], \end{array}$$

then this operator has the distinctive property that the inequality in equation (3.35) is satisfied in the domain of integration.

Using $L_{k_{R},k_{I},\xi }$ and following similar steps to those used in proposition 3.2, we can derive the result below.

**Proposition 3.5.**

Given the complex-valued function $q_{0}(x,y,z)$, *whose* $L_{1}$ *and* $L_{2}$ *norms are sufficiently small, let* $\mu_{0}(x,y,z,k)$ *denote the solution of equation (3.2), where* q *is replaced by* $q_{0}$. *Let* ${\hat{q}}_{0}(\xi ,k)$ *be defined by the right-hand side of equation (3.11), with* q *and* μ *replaced by* $q_{0}$ *and* $\mu_{0}$, *respectively. Let* μ(x,y,z,t,k) *be the solution of the linear integral equation*

3.38a $$\mu (x,y,z,t,k)=1-\frac{1}{\pi }L_{l_{R},l_{I},\xi }\left[E\frac{\xi {\hat{q}}_{0}(\xi ,k)\mu (x,y,z,t,l+i\xi )}{l-k}\right],$$

*where*

3.38b $$E:=E(x,y,z,t,l_{R},l_{I},\xi )=\exp[iTt-3l_{R}\xi (\xi +2l_{I})t+i\xi x+2il_{R}\xi y+i\xi (\xi +2l_{I})z].$$

*Let* q(x,y,z,t) *be defined by*

3.39 $$q(x,y,z,t)=2\frac{\partial }{\partial x}\mu_{1}(x,y,z,t),$$

*where* $\mu_{1}$ *is the coefficient of* 1/k *in the large* k *expansion of* μ. *Then*, q *solves the 3 + 1 equation*

3.40a $$q_{t}-\frac{1}{4}q_{xxx}-\frac{3}{4}\partial_{x}^{-1}(q_{yy}-q_{zz}+2iq_{yz})+\frac{3}{2}qq_{x}=0,$$

*with*

3.40b $$q(x,y,z,0)=q_{0}(x,y,z).$$

**Remark 3.6.**

In the linear limit μ→1, the above result gives rise to the following novel transform pair:

3.41a $$\hat{q}(\xi ,k)=\frac{1}{16i\pi^{2}}\int_{R^{3}}\;e^{-i[\xi x+2k_{R}\xi y+\xi (\xi +2k_{I})z]}q(x,y,z)\;dxdy\;dz$$

and

3.41b $$q(x,y,z)=\frac{2}{\pi }L_{l_{R},l_{I},\xi }[\xi \;e^{i[\xi x+2k_{R}\xi y+\xi (\xi +2k_{I})z]}\hat{q}(\xi ,k)].$$

This pair is the proper pair for solving the linear version of equation (3.40*a*), namely the equation obtained by neglecting the term $qq_{x}$.

It is interesting that equations (3.41) imply that $\hat{q}(\xi ,k)$ *automatically* satisfies the condition

3.42 $$\hat{q}(\xi ,k)=0,\;\text{for}\;k_{R}\xi (\xi +2k_{I})<0.$$

The easiest way to prove equations (3.41), as well as to derive equation (3.42), is to use the change of variables $k_{1}=\xi ,k_{2}=2k_{R}\xi ,k_{3}=\xi (\xi +2k_{I})$. Then, defining q(x,y,z) by the right-hand side of equation (3.41*b*), it immediately follows that $\hat{q}(\xi ,k)$ is given by the right-hand side of equation (3.41*a*). Equation (3.42) is a direct consequence of the Fourier transform pair bijection, where the role of the usual direct and inverse transforms is now reversed.

**Remark 3.7.**

It is important to note that a given evolution PDE often imposes an appropriate constraint that needs to be satisfied by the initial datum. A specific such constraint for KPI is analysed in [35]. Such constraints are, of course, ubiquitous for initial-boundary value problems reflecting the compatibility of the solution and its derivatives at the intersection of the spatial and temporal domains. Interestingly, for the case of the evolution PDE (1.1), the proper constraint is given by equation (3.42). What happens if the given initial datum violates this constraint? In this case, it is expected that there will be a singularity at t=0 (for a specific example involving initial-boundary value problems, see [36]). The analysis of this singularity is work in progress.

## 4. Conclusion

Equation (1.1), where q(x,y,z,t) is a complex-valued function and x,y,z,t are real independent variables, provides an integrable generalization to three-spatial dimensions of both the KP equations. Indeed, if $\partial_{y}=0$ or $\partial_{z}=0$, the t-independent part of the Lax pair of equation (1.1), namely equation (3.31*b*), reduces to the corresponding t-independent part of the Lax pair of KPI or KPII, respectively. Equation (1.6) provides a 3 + 2 generalization of the KP equations. Propositions 3.2 and 3.5 summarize the main results of this paper, namely they present a non-local d-bar formalism for solving the initial-value problem of these multi-dimensional equations. The unexpected achievement of the results of proposition 3.3 is the derivation of a formalism capable of dealing with a time dependence of the spectral function whose exponential contains a non-vanishing real part. Remarkably, this has implications beyond the area of nonlinear integrable PDEs: it introduces a new powerful transform for solving a large class of linear PDEs. It is worth noting that earlier modifications of the Fourier transform, such as those used by the author for the solution of boundary value problems on the half-line or the finite interval [37], involved the straightforward restriction of the direct Fourier transform in the appropriate *physical domain*. Here, the new transform involves a highly non-trivial modification in the *Fourier space*, thus, the new transform is indeed novel.

It is important to emphasize that the new formalism introduced in this paper for integrating equation (1.1) can also be applied to other multi-dimensional nonlinear PDEs, such as equations (2.3). This will be presented in future publications.

Several questions remain open. The most important among them is whether the 3 + 1 or 3 + 2 nonlinear PDEs introduced in this paper can be used to model physical or biological phenomena. In addition, in the formalism presented in both propositions 3.2 and 3.5, it was assumed that certain norms are sufficiently small. This excludes soliton-type solutions. The question of using such a solution for the scheme presented here remains open. Finally, the analysis of the singularity occurring at t=0 in the case that the initial datum does not satisfy the constraint in equation (3.42) is work in progress.

## Data Availability

This article has no additional data.
